# Heterogeneous LoRa-Based Wireless Multimedia Sensor Network Multiprocessor Platform for Environmental Monitoring

**DOI:** 10.3390/s19163446

**Published:** 2019-08-07

**Authors:** Sebastián García, Diego F. Larios, Julio Barbancho, Enrique Personal, Javier M. Mora-Merchán, Carlos León

**Affiliations:** Department of Electronic Technology, Escuela Politécnica Superior, University of Seville, 41011 Seville, Spain

**Keywords:** wireless sensor networks, energy efficiency, autonomous systems, environmental monitoring

## Abstract

The acquisition of data in protected natural environments is subordinated to actions that do not stress the life-forms present in that environment. This is why researchers face two conflicting interests: autonomous and robust systems that minimize the physical interaction with sensors once installed, and complex enough ones to capture and process higher volumes of data. On the basis of this situation, this paper analyses the current state-of-the-art of wireless multimedia sensor networks, identifying the limitations and needs of these solutions. In this sense, in order to improve the trade-off between autonomous and computational capabilities, this paper proposes a heterogeneous multiprocessor sensor platform, consisting of an ultra-low power microcontroller and a high-performance processor, which transfers control between processors as needed. This architecture allows the shutdown of idle systems and fail-safe remote reprogramming. The sensor equipment can be adapted to the needs of the project. The deployed equipment incorporates, in addition to environmental meteorological variables, a microphone input and two cameras (visible and thermal) to capture multimedia data. In addition to the hardware description, the paper provides a brief description of how long-range (LoRa) can be used for sending large messages (such as an image or a new firmware), an economic analysis of the platform, and a study on energy consumption of the platform according to different use cases.

## 1. Introduction

The study of environmental parameters and how these are changing related to human presence are a growing interest nowadays [1]. These studies are allowing scientists to predict the effects that this phenomenon (e.g., climate change) could have on our ecosystem [2,3]. The science of phenology studies how the different changes in weather affect animals and plants [4]. Phenology studies range from animals’ migration to plants’ flowering periods and how these phenomena are related to weather environmental conditions. In order to do that, the observation and recording of environmental, climatological, and multimedia data taken locally for long periods of time are essential.

However, these kinds of studies have been traditionally done by hand, which is tedious [5]. Usually, researchers and biologists have to go to the area to monitor; acquire information locally; and, after that, analyze this information manually (most common situation) or using automatic systems [6], generally designed for a specific application.

Furthermore, natural sites may have very restrictive access policies in order to preserve vegetation and wildlife from the stress of human interaction. Thus, the more autonomous and the less invasive the monitoring system, the better it is for the natural environment [7].

Wireless sensor networks (WSNs) [8] have been a great step forward with this problem [9]. This technology has allowed scientists to do their research in a more automatic and efficient way. Typically, in WSNs for environmental monitoring applications, sensors have gathered scalar parameters (temperature, relative humidity, solar radiation, and so on). Although these parameters provide interesting information to biologists and scientists, there are some research fields, like phenology, that require the capture of more complex information like sound and images (direct observation), which is not captured by traditional WSNs.

In order to cover the lack of sound and image data, biologists have specific multimedia capture devices available. Camera traps have been widely used by biologists [10,11] to acquire environmental images. Autonomous audio capture systems have also been proposed by some authors, but these systems still require moving locally to collect the data captured [12], or are wired-based [13].

A particular class of WSN are wireless multimedia sensor networks (WMSNs), which are able to retrieve multimedia data (audio and/or video) [14] in an autonomous way. However, video and audio signals have higher requirements than the traditional scalar parameters captured by WSN.

Multimedia signals need higher processing requirements in which other kinds of constraints are implied, for example, more power consumption [15]. Furthermore, multimedia monitoring may require a high use of the radio transceiver, which requires a high amount of energy to send multimedia data to a central processing system.

In addition, considering the nature of the application, the availability of large sources of energy is restricted. The most common situation in environmental monitoring scenarios is that the devices must be self-powered with energy harvesting systems like solar panels. Thus, energy management in WMSNs becomes even more important than in traditional WSNs used in environmental applications owing to the higher power requirements of this kind of sensor network.

Furthermore, WMSN platforms [16,17,18,19,20,21,22] only have image or/and audio sensors and do not include climate or meteorological sensors, which are also very important input parameters in environmental monitoring studies.

Moreover, these environmental monitoring devices are usually ad hoc hardware solutions designed for specific applications [23]. Therefore, the devices can hardly be used in other scenarios. Additionally, WMSN nodes have a complex internal firmware typically developed for specific applications and are hardly upgradable once they have been deployed, requiring going to the location, which could be arduous and complex.

Taking all this into account, this paper is focused on the design of a new flexible, scalable, upgradable, and reliable architecture for WMSN node to obtain and study environmental parameters, in an unattended manner, and with very low power consumption and high computational capabilities. In order to maximize computing capabilities, but also optimize the energy consumption, the node combines two independent processors: a low power microcontroller (LPM) and a high-end multimedia processor (MP), capable of executing local multimedia processing. In this sense, the proposed node is equipped with several multimedia interfaces, such as a visible camera, a thermal camera, and an integrated interchip sound (I^2^S) audio connection, which are complemented with climate and meteorological sensors. Finally, as radio communication interface, the node uses long-range (LoRa ) communications.

The main goal of the proposed architecture is to allow to dynamically determine the best tradeoff between power consumption and computational capabilities, adjusting the different hardware resources according to the needs.

The proposed hardware solution, which is freely available as open-hardware in the work of [24], aims to solve the main constraints that WMSNs have in environmental monitoring applications; that is, computational resources, communications, energy consumption, and sensors management. We encourage people conducting research about these topics to use the proposed platform.

The design is expected to be a multipurpose solution to reach different environmental monitoring applications, such as phenology, wildfire prediction [25] and detection [26], animal monitoring [27], and so on.

As proof of the advantage of the proposed design, an energy comparison between two of the main data processing models in WMSN has been carried out—a local processing, as is the case with the proposed nodes, versus a centralized processing, in which the nodes have to stream multimedia signals (which could be compressed or not) through the radio module.

The paper that is going to be presented is not just theoretical, but it is supported by several deployments in the Doñana Biological Reserve (Andalucía, Spain) in which the different solutions have been applied, and where the proposed node has been fully working since 2018.

The rest of this paper is organized as follows. Section 2 gives an overview of the main problem that WMSN’s nodes have in environmental monitoring. Section 3 introduces the proposed node architecture and the different functionalities that must solve some of the actual problems of WMSN for environmental monitoring. A study case of energy consumption of edge processing and multimedia streaming for a centralized processing is discussed in Section 4. A comparative result analysis of the previous study case is provided in Section 5. Finally, the conclusions are presented in Section 6.

## 2. WMSN Hardware State-of-the-Art and Their Limitations in Environmental Monitoring

In this chapter, the four main constraints and problems associated with WMSN applied to environmental monitoring will be analyzed. These main issues/constraints are related to computational resources, communication, sensor management, and energy consumption. However, as will be seen along this analysis, all these constraints are related to each other, meaning that a tradeoff between them must be found.

### 2.1. Computational Resources

Taking into account that WMSNs have to deal with more complex sensors than typical WSN, computational resources become a necessity in these kinds of sensor networks. The use of architectures with medium and high processing capabilities is required, as well as the availability of low power consumption modes with which to put the system in an idle state.

On the one hand, few available WMSN platforms use systems with a single, medium performance processor [18,19,21]. However, these medium performance architectures have very limited resources in terms of memory sizes (a few megabytes) and computational resources, which makes the development of complex multimedia processing applications difficult. Furthermore, even some of these processors have low power modes. Their consumption cannot be discarded. Therefore, having such a kind of processor, the tradeoff between computational resources and consumption becomes a non-trivial task.

On the other hand, there are some architectures that propose systems with more than one processing device [16,17,20,22]. These architectures have a hybrid computational schema: a high-end device (typically a single instruction, multiple data (SIMD), field-programmable gate array (FPGA), etc.) and a low-end device (typically a general-purpose microcontroller) in the same node. Specifically, real-time pre-treatments of multimedia signals can be done with the high-end device. Conversely, the low-end device is used to perform specific data processing and/or node management tasks. With these hybrid architectures, the different systems in the node must be managed carefully as the high-end devices are power-hungry.

However, most of these hybrid solutions are very specific for a task and cannot be easily used for another scenario, or to improve its capabilities. Moreover, common proposed high-end devices are complex to program, as they are very application focused, not allowing the use of, in many cases, standardized libraries and programing techniques.

### 2.2. Communications

Unlike traditional WSNs in which the volume of generated data is low, WMSNs generate a higher volume of data because of the more complex sensors. Traditionally, in WSNs, the low volume of data captured by the nodes allows them to send the raw data through the wireless communication, but in WMSNs, the transmission of the raw data becomes more difficult.

Transmitting such large volumes of data requires a high communication bandwidth. Taking into account that the higher the communication bandwidth, the higher the consumption and the lower the radio range, the transition of multimedia data is very difficult in WMSNs used in natural scenarios. Furthermore, transmitting via radio large volumes of data (whatever the bandwidth used) increases the consumption in a drastic way, as the radio module is one of the main sources of consumption in a node [28].

Furthermore, deploying sensors in natural environments means that the range of the devices must be as high as possible, because these sites are large spaces in which most of the parameters have negligible variations in short distances. In this sense, IEEE 802.15.4 [29] has been the most common standard communication protocol used by environmental WSNs [30], but it has certain limitations in terms of its range, with just around 200 m in line of sight. However, new radio technology like LoRa [31] is opening new possibilities to wireless communication. LoRa is a wireless communication standard thought to low power and wide area applications (LPWANs) with a range of 10–15 km [32], but with a lower bandwidth than IEEE 802.15.4. This lower bandwidth may be a problem for multimedia streaming, but not for traditional WSNs for environmental monitoring.

As a conclusion, applications in natural areas require radios with a high range as well as with a low power consumption, but these kinds of radio technologies usually have very low bandwidth. Therefore, in WMSNs for natural application, the communication requires a tradeoff between versatility and power consumption that, until now, has not been well solved.

### 2.3. Sensors

The most common sensors used in WMSNs are microphones and cameras, but scientists also need other kinds of sensors for their research. Some platforms just have cameras, others just microphones, and there are also a few platforms that have both. However, WMSN platforms traditionally do not include other kinds of environmental sensors. Image and audio are great input parameters for biologists, but sometimes, for some kinds of research, other parameters like meteorological data are also needed. Moreover, the addition of multimedia and meteorological sensors may have certain implications.

On the one hand, meteorological sensors do not have the complexity that multimedia sensors have. In this sense, in traditional WSNs, low power microcontrollers with limited resources have been used.

On the other hand, however, as it has been said, multimedia sensors require higher computational requirements, which also have higher consumption. Furthermore, multimedia and meteorological data may have different sampling rates depending on the specific application.

Thus, dealing with different kinds of sensors may require different approaches in terms of microcontrollers, sampling rates, energy management profiles, and so on. To deal with this problem, some authors propose multi-tier networks [33] in which two or three kinds of node architectures are used in the same sensor network. This methodology allows us to overcome the sensor limitations, but increases the development complexity, because of its requirement of the integration of very different nodes into the same network.

### 2.4. Energy Consumption

As it was said, energy consumption is a key factor in environmental monitoring because the devices are typically installed in a remote location with no available energy sources. To solve this problem, environmental devices are usually powered by batteries and/or solar panels. In this sense, environmental sensors must be energy-autonomous devices in which a strict energy management system is required.

The conjunction of more complex sensors, higher volumes of data, higher computational requirements, the nature of the location, and so on makes energy consumption one of the main topics to take into account in WMSNs applied to environmental monitoring, being a problem not well solved yet.

The radio has traditionally been the main consumption element in WSNs. The higher volume of data generated by multimedia sensors makes it quite inefficient in terms of energy consumption of the transmission of these data through the radio module. To solve this problem, some authors propose data compression algorithms [34,35,36] as well as data fusion algorithms [37] to reduce data traffic in the network and, therefore, the energy consumption. Other authors have proposed different communication protocols [38] and routing mechanisms [39] with the same final objective: reduce energy consumption in WMSNs.

Although the presented solutions are valid for certain applications, the sensor nodes would still send more information than is really needed by biologists, and they would have to analyze large volumes of data. In this sense, other proposed platforms take advantage of edge processing [40] in order to reduce the use of the radio transceiver, sending just the result of the processing [41].

However, note that all these solutions to reduce communication volume require high computational resources in order to do the compression and/or the local data processing. Thus, the communication affects the computational requirement, which also affects energy consumption. Once again, the different constraints in WMSNs are related to each other.

Although all these papers propose software-based methods, the whole hardware of a WMSN node must be energy efficient [42]. In this sense, WSN and WMSN hardware platforms are constructed with low power components trying to minimize their power consumption. Traditionally, WSN power management has been done combining hardware sleep modes with radio transmission strategies. In the same way, in WMSNs, different radio transmission schemas have to be used to reduce energy consumption. Hardware sleep modes have also been used in some WMSN platforms but, taking into account that the devices typically used in these platforms are high-end devices not usually designed for a low power operation, there may not be low power modes available, or not ones with a consumption as reduced as needed in environmental monitoring. As an example, platforms like CITRIC [17], which has a high-end processor with embedded Linux, has a consumption of around 450 mW in its lower power mode which is a quite high value for a device that needs to be powered with a small solar panel. Thus, the hardware must be carefully designed in order to have the minimum consumption possible.

## 3. Proposed WMSN Node Architecture

As a summary of the previous section, it is easy to highlight that nowadays, there are some constraints in WMSNs that make its application complex in environmental monitoring applications. There are platforms that solve some of these problems separately, but none of them can be used as a generic platform to deal with all the main issues faced in environmental monitoring.

**Figure 1:**
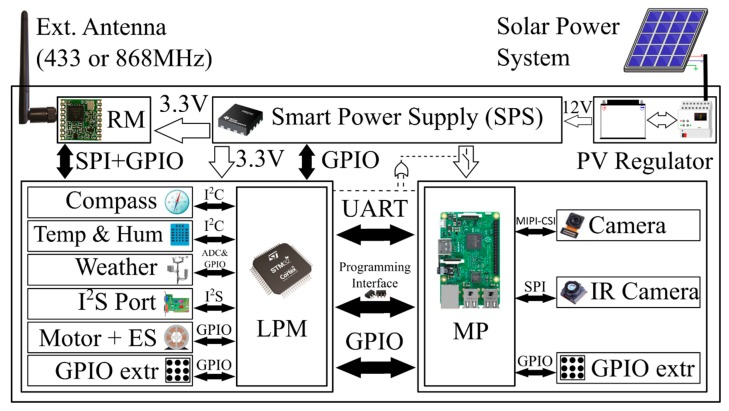
Node architecture. LPM, low power microprocessor; MP, multimedia processor; RM, radio module.

Therefore, this paper proposes a new node architecture for WMSNs designed to deal with these problems. The main target of the developed node is to obtain a robust, flexible, and scalable system that has a solution for most of the identified constraints associated with capture systems for environmental monitoring applications. The complete design of the multimedia node is broken down into different sub-sections, as well as their main characteristics in terms of processing, consumption and energy management, sensors, and communications. The general node architecture can be seen in Figure 1.

### 3.1. Hardware Architecture

Taking into account the different constraints that WMSNs have in terms of energy management and processing capabilities, a multiprocessor architecture was chosen in order to maintain a balance between energy consumption, state of charge, and computing capabilities. This architecture makes use of edge processing, which, as it will be seen, remarkably improves battery life time.

The main parts of the node are as follows:

- *Low Power Microprocessor (LPM):* The low power microprocessor is the manager of the node. The LPM is based on an STM32L162RD, which is an ultra-low-power ARM (advanced reduced instruction set computer, RISC, machine) Cortex-M3 processor that combines good performance with very low energy consumption. This microcontroller has, among others, 384 KB of Flash, 48 KB of RAM, 12 KB of EEPROM, a 12-bit analog to digital converter (ADC), AES encryption by hardware, I^2^S audio interface, real time clock (RTC), and direct memory access (DMA). Currently, this device is state of the art in power efficiency in microcontrollers. Even maintaining the power consumption very low, its computational capabilities are several times higher than the classical devices used in WSNs.
- *Multimedia Processor (MP):* Based on a Raspberry Pi 3, the MP acts as a slave of the LPM being just another resource at his disposal. The Raspberry Pi 3 is based on a Broadcom BCM2837 processor with an ARM Cortex-A53 with a quad-core architecture, up to 1.2 GHz, digital signal processor (DSP) and SIMD instructions with NEON technology, and a dual-core graphics processing unit (GPU) coprocessor VideoCore IV. This device was chosen because it provides a good tradeoff among cost, power efficiency, and computing capabilities. There are other alternative more efficient or more powerful ARM-Linux devices, but at higher prices, and not suited for large networks deployments.
- *Multimedia Sensors:* As image sensors, the node has a CMOS 8MP (3280 × 2464 pixels) visible camera based on the Sony IMX219PQ IC (connected via a mobile industry processor interface -camera serial interface, MIPI-CSI) and a low resolution (180 × 120 pixels) infrared (IR) thermal camera based on the forward looking infrared (FLIR) Lepton 3 (connected via a serial peripheral interface, SPI). Both image sensors are controlled by the MP. As audio sensors, the node does not include by default any microphone, but an I^2^S connection is available on the board to connect any audio sensor to the LPM. Alternatively, a USB soundcard can also be connected to the MP.
- *Traditional Sensors:* The node is equipped with traditional scalar sensors. Temperature and humidity can be obtained through an I^2^C (inter-integrated circuit) sensor based on the HTU21D IC. The node also has an additional connector for a weather station, which includes a wind vane, an anemometer, and a rain gauge.
- *Power Supply:* The device is meant to be used with energy harvesting systems (typically a photovoltaics, PV, solar panel with a regulator and a battery). It is designed to work in a range of 7–18 V. A dual power supply with two different power rails was implemented: 3.3 V and 5 V. The 5 V rail can be activated or deactivated, allowing one to cut out the energy to the power-hungry devices of the node: MP and cameras. The power supply is managed by the LPM, but, in some cases (reprograming task), the MP can also take the control of it. The power supply was designed with switched technology integrated circuits (ICs), MAX5033 and NCP3170B, which have a very high efficiency (typically in the 85%–95% range). As will be seen in future sections, the power supply management significantly increases the battery lifetime.
- *Radio Module (RM):* Radio communication is based on the LoRa radio communication using the 868 MHz free Europe band. A module with the SX1276 Semtech LoRa IC was used. The RM is also managed by the LPM. As has been depicted before, this radio has a high range and low power consumption with a limited bandwidth, but, because of the use of the MP with local processing, we can use it in a WMSN node. This module is completed with a subminiature version A (SMA) connector for a better location of an external antenna.
- *Others:* A stepper motor interface and its endstops (ESs) is also provided to add a rotate system for the node to cover the maximum field of view (FoV) possible with the cameras, reducing the number of nodes required to monitor an area. A digital IC compass based on the LIS2MDL is also included to know the actual orientation of the node. Additionally, a joint test action group (JTAG) programming interface is shared between the LPM and MP, giving the node auto-reprogram capabilities. Also, a general purpose input/output (GPIO) as input/output (IO) expansion connector was made available with plenty of interfaces for both processors to connect any other sensor that any specific application may require. Therefore, the proposed node is easily adaptable to different environmental monitoring scenarios. A communication interface is shared between the MP and the LPM.

**Figure 2:**
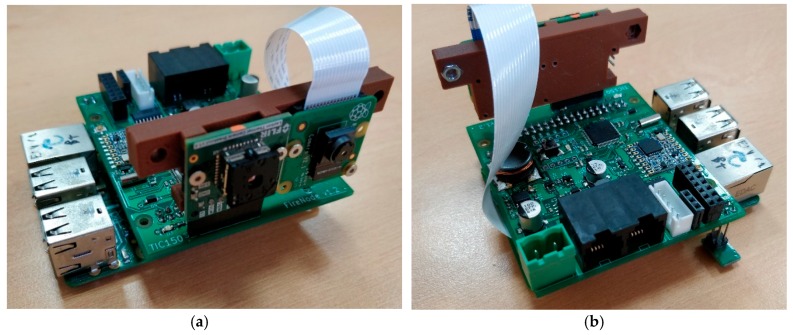
Hardware of the proposed node: (**a**) front view of the node with the thermal camera on the left and the visible camera on the right; (**b**) back view of the proposed node with the connectors.

**Figure 3:**
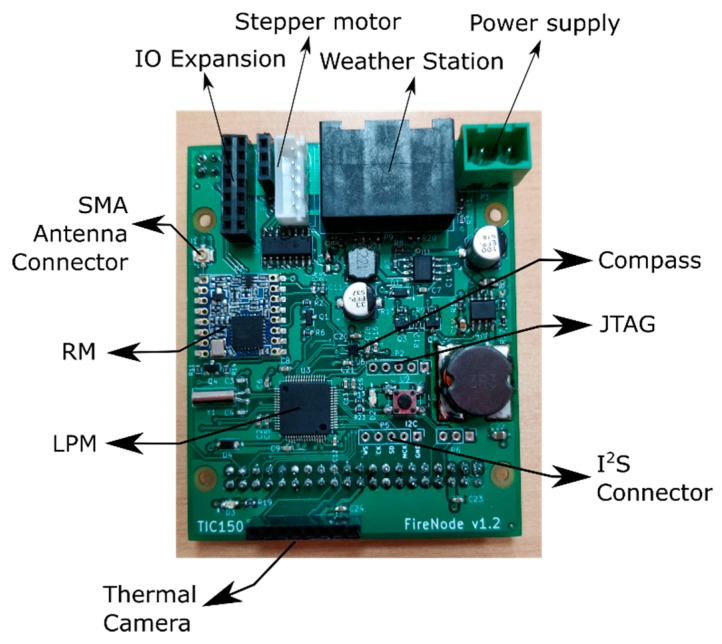
Different parts of the custom-based printed circuit board (PCB).

Physically, the node was constructed using two stacked boards (Figure 2): a commercial off-the-shelf (COTS) Raspberry Pi 3 board and a custom-based board (Figure 3). The custom-based board has all the cited components apart from the Raspberry Pi. The proposed node could be used also in multi-tier network schemes, using one tier with just the custom-based board acting as a traditional WSN node and another tier acting with all the components as a full WMSN node. Also, depending on the application, the thermal camera can be included or not in the final application.

Moreover, currently, the pinout of the Raspberry Pi has become the standard among different ARM-Linux based boards. Therefore, the proposed system can be easily updated with more powerful devices if the application requires greater computational resources, but, obviously, with an extra cost in prize and power consumption.

In this sense, this platform offers the advantages of hybrid and multi-tier networks, but in a more integrated way, reducing the complexity of the development of a network.

The STM32L162 (which acts as the LPM) is an ultra-low power microcontroller that is used to acquire low-bandwidth sensor information. For this purpose, all the scalar sensors were connected to the LPM. All these sensors have low-power modes (compass, temperature, and humidity sensors) or have non-active electronics (weather station). This acquisition can be performed with low-power consumption, as will be seen. Additionally, the platform used for the LPM is based on an ultra-low power ARM Cortex-M architecture, which is more powerful than traditional 8-bit architectures used in other platforms [16,17,20,22].

The Raspberry Pi (which acts as the MP) is used to acquire and process data either from the image sensors or other data sources (audio coming from the I^2^S sensor connected through the LPM, data from other nodes, and so on). The Raspberry Pi is a power-hungry device that is not designed to operate in a low-power mode, having a considerable energy consumption in stand-by for environmental applications. The cameras also have high energy consumption and, as well as the Raspberry Pi, they have non-discarded energy consumption in its low-power modes. For this purpose, the LPM can control the power supply associated with these components, completely switching off these elements when they are not needed. Additionally, it needs to be said that the platform used for the MP is currently a state of the art in embedded high-end processors based on an ARM Cortex-A architecture, which is more powerful than ARM7 architectures used in other platforms [18,19] or PIC32 architectures [21].

The LPM and the MP have a shared universal asynchronous receiver-transmitter (UART) interface with which the LPM can assign tasks to the MP. As will be shown in the processing scheme, the LPM is able to evaluate the conditions in which the MP is needed to run and when it does not, allowing the node to be more efficient. This interface is also used to share data between the two processors (algorithms results, configurations, multimedia data, and so on). Moreover, a set of GPIO pins are also shared between the LPM and the MP.

**Figure 4:**
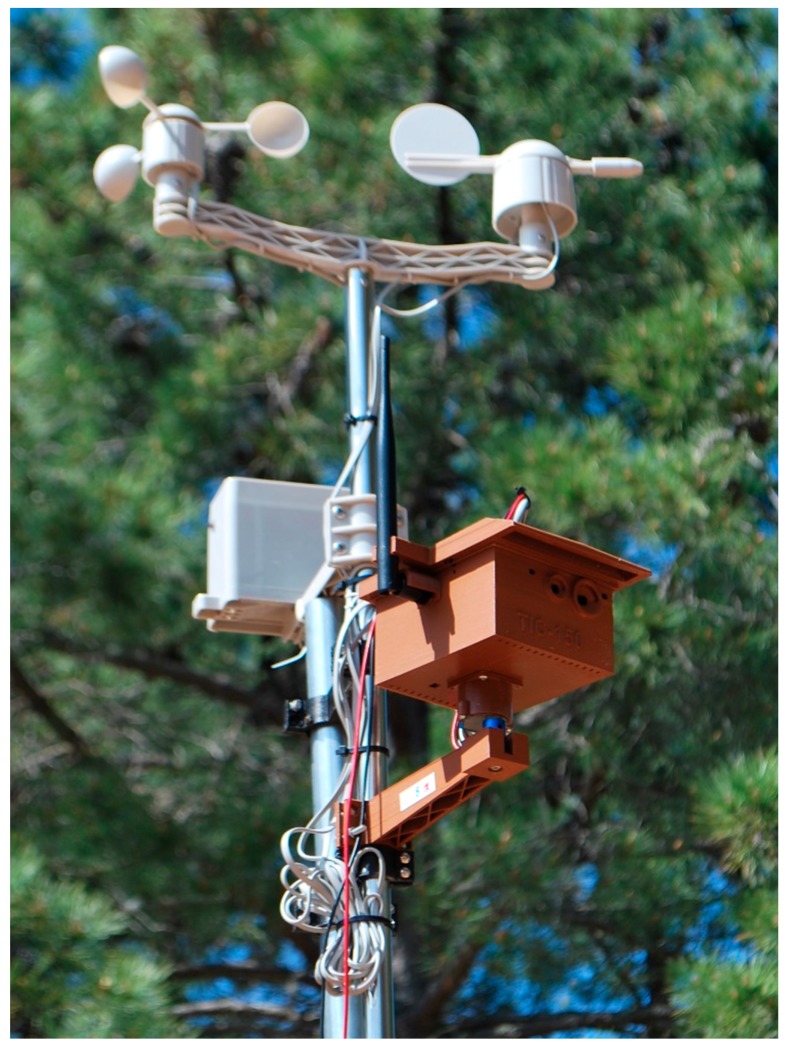
Proposed node fully assembled and deployed in the Doñana National Park.

In Figure 4, the completely assembled node (electronics, antenna, stepper motor, enclosure, weather station, and so on) is shown. The enclosure was designed using 3D printing technology using acrylonitrile butadiene styrene (ABS) plastic. The enclosure has proven to be resistant in the wild—proof of this is that a network was deployed in the Doñana National Park using the proposed devices (Figure 4).

Specifically, this network was made up of 10 nodes, covering an area of 40 Ha for an application of fire detection, working continuously throughout a year with no issues.

This platform is designed to improve the constraints of WMSN listed in Section 2. In this sense, the proposed platform offers a great tradeoff between computational resources and consumption thanks to the developed architecture. Additionally, the proposed platform can be programmed at a high level using different programming languages and libraries thanks to the Raspberry Pi. Furthermore, thanks to the high integration of sensors that the platform has, the device can be used for different applications and scenarios having a multipurpose environmental monitoring platform.

### 3.2. Processing Scheme

To keep the power consumption low, the design of a smart device is not enough, a software design that allows one to maximize the efficiency of the architecture is essential too.

Therefore, the software developed is driven by the need to reduce power consumption without compromising the node performance, and following the main goal of extending battery lifetimes.

**Figure 5:**
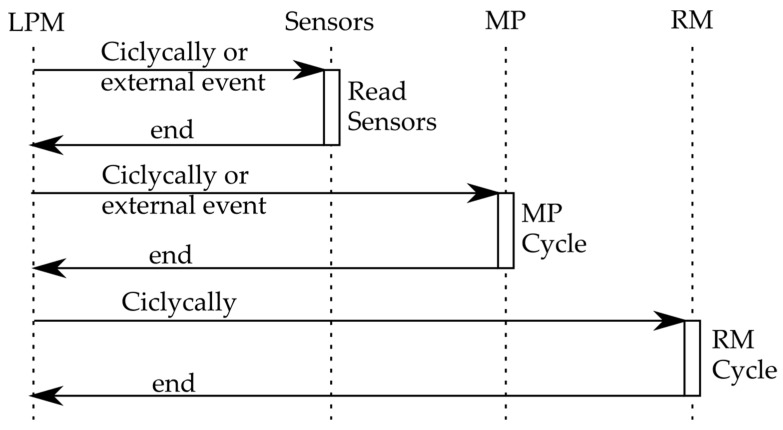
Processing schema of the proposed node.

For this reason, all the software implementation needs to be designed based on hibernation (on/off) cycles, which are controlled by LPM. This manager is designed to mainly deal with three different tasks (Figure 5):

1. Cyclically, or based on external sensor information, the LPM acquires environmental information and stores it in a temporary storage area.
2. The LPM wakes up the MP cyclically or based on external events in order to execute some task according to the needs of the system. In this sense, and based on the application, the LPM is responsible for controlling the MP cycles based on the state of charge and the current needs.
3. Cyclically, the LPM wakes up the RM to attend to its reception window and (if necessary) informs other nodes or the base station (BS) of relevant information.

When the LPM has no pending task to perform as the energy manager of the node, it can change its state (and the rest of the elements of the node) in its low consumption mode, waiting in this state until the next wake-up cycle is reached.

One key feature of the proposed architecture is that the MP is acting as a peripheral to the LPM. This allows the LPM to dynamically use the MP as needed. As described before, the LPM can wake-up and shut down its own peripherals and the MP to execute tasks according to the different needs of the system. When the MP is in ON, a command from the LPM tells the MP which action is needed.

Having the LPM as the manager of the node could significantly improve battery life. As was said, this manager can be programmed to dynamically change the MP cycles according to the state of charge and the specific application. For instance, if the device is used to predict wildfire, a raining day is not the most likely day for there to be a fire. Moreover, a rainy day may not be the best for the solar charging system. In this sense, the LPM (based on the weather data) can be programmed so that on rainy days, it does not capture multimedia data with the MP, which requires high energy consumption. The opposite strategy can be applied if there is a warm day, with not much humidity and high winds.

Actions of the MP can be divided, basically, into three main tasks: (1) collecting data and processing it, (2) sending data to the LPM (in order to send it through the RM) either from RAM or its internal storage, and (3) reprogramming tasks. When the MP has finished all the requested tasks, the LPM sends a shutdown command to the MP and later to the power supply.

The collection and processing of data done by the MP is divided into the following sub-tasks:

1. Firstly, the MP captures data from multimedia sensors. Depending on the application, different kinds of multimedia sensors may be used. This acquisition stage may require retrieving audio data from the LPM I^2^S audio sensor.
2. After acquiring multimedia data, and depending on the application, the MP may require data from the traditional scalar environmental sensors connected to the LPM in order to do some correlation with the multimedia information.
3. Once the MP has all the data the application needs, its local processing is done.
4. Relevant information can be stored in the SSD (solid-state drive) if necessary. Thanks to this ability, the MP can store the results of the local processing as well as other kinds of data (sensor values, processed multimedia data, and so on) to future uses.
5. The results of these local MP processing algorithms are sent to the LPM, which, in the case of a relevant result, transmits it to other nodes or to the BS. In this report, if any multimedia data have been saved in the MP internal memory, a reference to these data is also shared by the MP, for a possible later request on demand.

As will be seen, this proposed scheme, in which local processing is done, improves battery life quite significantly compared with multimedia streaming in most cases, even using compression methods. Although, in exceptional cases, multimedia data can be also sent through the radio module.

For this reason, the MP is able to retrieve data to the LPM, which is the last one responsible to resend it through the radio module. Taking into account the limitations in terms of RAM size of the LPM, the multimedia data retrieval is done by the MP, segmenting the data into several packets that are requested by the LPM. The LPM is able to request any fragment of the data to the MP.

Other important tasks that can be done by the MP are reprogramming tasks, which will be explained later in a separate section.

The ON/OFF cycles of the MP must be carefully handled by the LPM, as the MP runs an embedded Linux OS. In the ON cycle, the LPM must know when the MP has finished booting and the application is ready to receive commands. Conversely, in the OFF cycle, when the MP has completely shut down the OS in order to shut down the MP’s power supply.

For the wake-up cycle, the LPM sends an on command to the power supply then, the LPM keeps waiting until a command comes from the MP indicating that everything is up and ready.

Also, for the off cycle, the LPM sends a command to the MP to turn off the Linux OS safely. Just before the MP is ready to turn off the OS, it sends a command to the LPM to indicate that its off cycle is going to start (the MP application is no longer active after this). The LPM, after receiving this command from the MP, starts a timer to give the MP time to completely turn off the OS. When the timer ends, the LPM sends a command to the power supply to switch off.

The LPM has different mechanisms to make the ON/OFF cycles and communication more robust. If no command has been received for a period of time (timeout) after the wake-up order indicating the correct or incorrect wake-up of the MP, the LPM makes one more attempt to power-on the MP. If the new attempt is not satisfactory, the LPM can send a report to the BS indicating the MP failure. Error commands can be sent by the MP indicating some error at the start-up (e.g., the camera cannot be initialized) or during operation (e.g., the LPM ask for an image retrieval that does not exist in the MP memory).

The LPM software was written in C, while the MP software was written in C/C++ running on a customized Linux OS to improve energy efficiency. As an advantage, the use of Linux allows the application of video and audio processing libraries like OpenCV [43] or STK (the synthesis toolkit) [44]. Therefore, the proposed platform facilitates the design of applications with the use of general libraries, despite common hybrid devices, which require programming very complex and specific devices.

### 3.3. Communication Scheme

**Figure 6:**
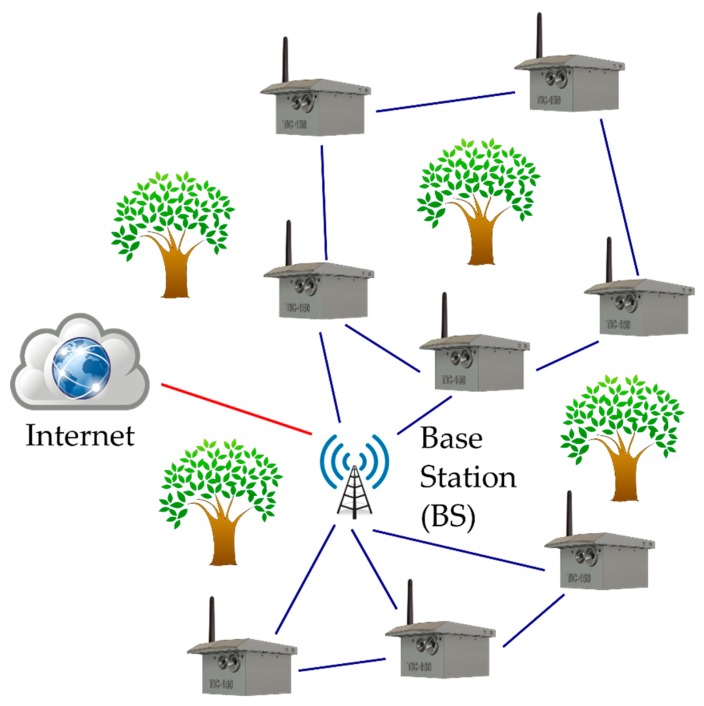
Communication schema used by the proposed system.

The proposed device works in a traditional sensor network schema in which a series of nodes is deployed around a base station, as can be seen in Figure 6. Nodes can share data between them in order to do cooperative processing, allowing it to balance the computational load among the network. The nodes communicate among themselves using the physical radio communications of LoRa [31]. In this sense, LoRaWAN [45] is not used in the upper layers as this protocol uses a star-based schema.

Thanks to the high radio range of the LoRa communication, it may not be necessary to add a multi-hop schema to reach the BS. Even so, the network can work in different topologies: star, tree, or mesh.

The BS in this schema acts as a data sink, collecting the messages received from the network. Moreover, the BS acts as a supervisor and coordinator, sending commands to the different nodes of the network. The physical device deployed in the field acts just a gateway between the nodes (edge) and the upper layers (fog and cloud). Thus, the logic of the BS is located in the fog or in the cloud layer of the network. In this schema, the BS must have an internet connection.

As described before, the communication among nodes and with the BS is based on LoRa radio transceivers. This radio has a large coverage, low power consumption, and a low data rate. Having large coverage and low power consumption makes this radio quite suitable for environmental applications, but its low data rate makes multimedia data streaming difficult [46]. However, considering the advantage of the proposed local processing, the devices are responsible for sending just the results of the local processing (only when necessary) and not large amounts of data.

Nevertheless, a way has been established to transmit large volumes of data using LoRa, as will be seen. However, it is quite inefficient in terms of energy consumption and radio channel occupancy (time of use).

LoRa uses the license-free 433, 868 (Europe) and 915 MHz (North America) bands. The proposed node works in the 868 MHz band, even though it could work in any of these bands just changing the RM module. This module can be changed during fabrication using the same PCB design.

In comparison with other proposed platforms, the use of LoRa instead of the IEEE 802.15.4 or WiFi drastically improves the radio range as well as the power consumption. Additionally, the use of a multi-hop scheme (currently not part of LoRaWAN specification) can improve the radio range even more.

Even though LoRa has a reduced data bandwidth, thanks to the proposed processing scheme, not a large amount of data needs to be transmitted, but rather just the result of the analysis (a few bytes).

### 3.4. Reprogram Capabilities

As has been explained above, the proposed architecture uses complex internal firmware, especially the MP, which carries with it most of the data processing algorithms. In addition, these devices are installed in natural sites, which are usually in remote locations, making it difficult and tedious to update the firmware. For this purpose, some authors have proposed different reprogramming procedures and code disseminations strategies at the edge in order to solve this problem [47].

Taking this into account, the proposed node can auto reprogram itself (both the MP and the LPM). New programs can be stored in the MP storage drive or can be sent over the radio link. The philosophy is that the MP can reprogram the LPM and vice versa. This is not common in typical WSN or WMSN platforms.

For this purpose, the UART interface can be used by the MP to reprogram the LPM. This can be done using the internal factory-programmed bootloader of the STM32L162, which can be enabled using the BOOT0 pin at the start-up of the microcontroller. To do that, the MP is able to control the reset and the BOOT0 pins in order to put the LPM in bootloader mode and reprogram it. In addition, the MP can also be reprogrammed, because, as the MP’s application runs on a Linux OS, the reprogramming simply consists of switching from executable files.

New programs may come from the internal storage of the MP or from the upper layers of the network transmitted via radio link. However, for the reprogram process as such, programs must be in the internal MP storage because the LPM may not have enough memory (RAM or Flash) to temporarily store it. Thus, when a command from the radio is received pointing out that an update is going to be sent, the LPM wakes the MP up and puts it in file reception mode. When the MP is ready, the LPM sends an acknowledgement to the BS, indicating that the node is ready to receive the new program. At this moment, the BS starts transmitting the new file. The transmission is done using redundancy checks to prevent data corruption. Additionally, alongside the new file, a redundancy code obtained from the entire file is sent. This redundancy code is checked after the file is saved to prevent any corruption in the whole transfer process.

The load of new programs for both the LPM and the MP is always commanded by the BS, so new programs can be uploaded to the memory of the nodes, but not burned/executed. The radio command to execute/burn a program contains what processor and which file is going to be reprogrammed.

For the MP reprogram, as has been said, it is simple, because the reprogram just consists of switching from executable. The process will be as follows:

1. After receiving the radio command, the LPM wakes the MP up and puts it in reprogram mode.
2. The LPM asks for the new program file. If found, the process continues; if not, an error is sent to the BS.
3. The LPM requests the MP to change the current application to the new one. This change would be valid after the reboot of the MP.
4. The LPM reboots the MP. When the MP is active, it sends the LPM a command indicating a successful start (the command is the current software version).
5. A command is sent to the BS indicating a successful update.

For the LPM, is a little bit difficult because it requires disabling it temporarily to put it into bootloader mode. As the LPM is the manager of the node, this action must be taken carefully. The process will be as follows:

1. The LPM wakes up the MP and puts it into reprogram mode.
2. The LPM asks for the new program file. If found, the process continues; if not, an error is sent to the BS.
3. The LPM hands over the power supply management to the MP. After that, the LPM orders the MP to reprogram it.
4. The MP resets the LPM and enables the bootloader mode (using the reset and the BOOT0 pin).
5. When the LPM is in bootloader mode, the MP reprogram its flash using the UART interface.
6. Once the programing has finished, the MP resets the LPM. When the LPM has started, it sends a command to the MP indicating a successful start (the command is the current software version).
7. The MP gives back the power supply management to the LPM.
8. The LPM shuts down the MP. A command is sent to the BS indicating a successful update.

### 3.5. Cost Analysis

One essential factor throughout the design of the proposed device was the cost—trying to reduce the final price of the system to an acceptable value. It is important to highlight that the indicated prices are for a short run. Having larger runs would make the system cheaper.

As has been stated previously, the device is very adaptable, depending on the application. In this sense, different parts of the node can be removed or added according to the application needs—the final price would be given by the parts used. For instance, if the environmental application just needs typical weather data, with just the custom-based PCB and the weather station (without MP, cameras, and so on), this would be a typical WSN node architecture. However, if a more complex sensor is needed with image, audio, weather sensors, and so on, the whole node architecture would be needed. Therefore, the first example would be cheaper than the second one.

In this sense, the different parts of the node were split into different parts; the node can be configured by adding or removing any of these parts. The node must always need the custom-based PCB as a base, which has the temperature and humidity sensors built-in as well as the LoRa transceiver.

Table 1 shows the different parts needed for the basis configuration of the node. Having this as a base, any of the next components of Table 2 can be added according to the application needs.

As an example, three possible configurations are shown in Table 3: (A) a typical environmental monitoring WSN node architecture, (B) a visual sensor network node, and (C) the complete proposed node (without the thermal camera).

In order to make a comparison, one of the most used in WSN applications is the Waspmote device by Libelium [48]. This platform has a cost of 220 €, but it just has a single-core 8-bit microcontroller based on the ATmega1281 (which is much less powerful than the LPM of the proposed platform), an accelerometer, and a LoRa radio transceiver. With this hardware configuration, its cost could be comparable to our “basic Node” configuration (see Table 1).

## 4. Study Case. Energy Consumption Estimation of Edge Processing and Data Streaming

In general, there are two approaches to image processing in WMSN: a centralized processing either fog or cloud computing [49,50] or local processing at the nodes (edge computing) [40]. This section describes a study case using the proposed platform of these two image processing approaches in WMSN. On one hand, an image capture, compression, and transmission energy analysis and, on the other hand, a local processing energy analysis. In Section 5, the results of these two approaches will be analyzed and compared.

### 4.1. Measuring Set-Up

**Figure 7:**
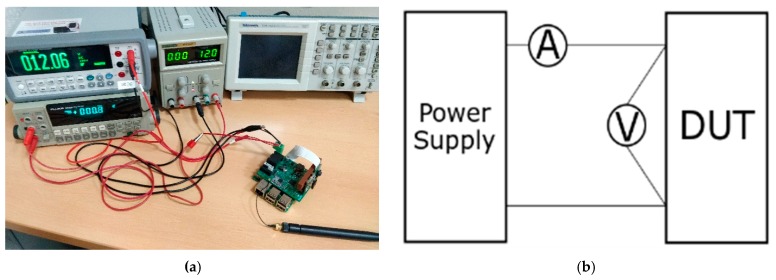
Measurement set up. (**a**) Image of the equipment used for the tests: two bench multimeters, a power supply and the proposed node acting as the device under tests (DUT); (**b**) measurement diagram.

In order to measure the power consumption of the proposed node, two bench multimeters were used: a Fluke 8842 A and a Keysight 34,450 A. The measuring set-up is shown in Figure 7.

Having the Fluke^®^ reading current, the Keysight reading voltage, and the proposed system acting as the device under tests (DUT), the power consumption and the energy of the proposed system can be easily calculated.

Both multimeters can be controlled and read remotely. For this purpose, a MATLAB^®®^ script was written to acquire and process the data. Also, the multimeters were synchronized to read both variables at the same time.

### 4.2. Capture, Compression, and Data Streaming (Fog Computing Approach)

Centralized processing has been the traditional approach in conventional WSNs. This approach requires streaming the data through the radio module to a central node, where the processing is done.

In this test, an image captured using the CMOS camera connected to the MP was transmitted through the radio module. In order to deal with the large volumes of data, some authors have proposed different compression algorithms to reduce transmission times (and so the energy consumption) [34,35,36]. Taking into account that, with this approach, the processing is going to be done after decompression, the image-compressing algorithm should not be aggressive in terms of losses, preserving a good image quality after decompression.

Usually, the more compressed an image is, the more losses it has, making the processing difficult after decompression. However, the more compressed the image, the shorter the transmission time (and the lower the energy consumption). Thus, a tradeoff between compression ratio and data loss must be met.

#### 4.2.1. Compression Algorithm

**Figure 8:**
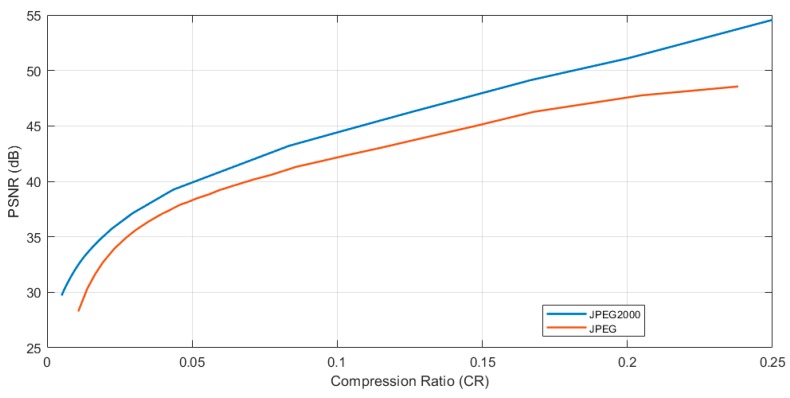
Peak signal-to-noise ratio (PSNR) and compression ratio comparison between JPEG2000 and JPEG.

For this study case, the JPEG2000 [51] compression algorithm was used. JPEG2000 offers both lossless and lossy compression provided by using a reversible integer wavelet compression. This algorithm has good results with raw images in terms of compression ratio and efficiency compared with others [52]. It also has a good peak signal-to-noise ratio (PSNR) in lower compression ratios compared with a typical join photograph expert group (JPEG) compression, as can be seen in Figure 8. This graph was obtained by running the algorithms on the proposed platform using a subset (landscape images captured with the D40 camera) of the RAISE raw image dataset [53].

As can be seen from this analysis, with the same quality after decompression, JPEG2000 offers a better compression ratio, which will result in lower transmission times. However, JPEG2000 has a slightly higher computation cost (in terms of time) than traditional JPEG, which will also affect energy consumption, as will be seen later. Although, it reduces the radio transmission times, which are more energy demanding.

#### 4.2.2. Data Compression and Streaming Considerations

Prior to this study, some things must be pointed out. As LoRa communication allows a maximum payload size of 255 bytes, the compressed image needs to be fragmented into packets. Taking this into account, it will be supposed that 16 bytes are used as headers, containing sender, receiver, next node (in case of multi-hoping), number of fragments, message type, an ID of the image, and a counter (to avoid error on repetition). In summary, 239 bytes of effective payload can be used.

The LoRa modem is configured to its maximum transmission rate (SF = 7 and BW = 500 MHz). The node used in this study works in the 868 MHz band.

The images were captured from the CMOS sensor of the MP at a resolution of 1280 × 960 pixels. Therefore, the size of a raw image was 3,686,400 bytes (three channels with one byte per pixel).

A PSNR value after decompression (or a compression ratio) must be chosen to execute the compression. As an example, an image compressed with a PSNR quality of 35 dB was used for this study case.

To make the results repeatable and considering that the compression rate between two captured and compressed images with the same PSNR may vary, the transmitted file should always have the same size for the same PSNR. Thus, the nature of the captured image does not interfere in the packets transmitted, making the results repeatable. In this sense, to display how the size of a different captured and compressed image by the MP can oscillate, a dummy file (with a fix size, obtained by multiplying the raw image size by the compression ratio of Figure 8) is used to evaluate this sending stage.

**Figure 9:**
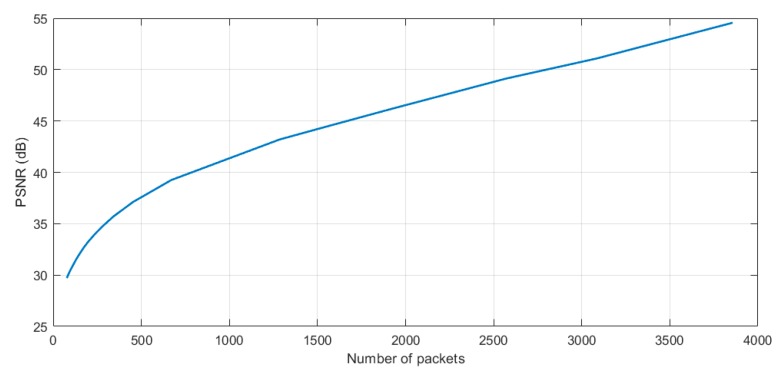
Packets for different PSNR values using JPEG2000.

As was commented above, this file must be fragmented into packets in order to send it through the radio module. Figure 9 shows the number of packets using all the available payload size (239 bytes) per different PSNR values using the JPEG2000 algorithm. The number of packets of Figure 9 was obtained by multiplying the size of a raw image per the compressing ratio obtained in Figure 8 per different PSNR values and dividing per 239 (number of bytes per packet).

Thus, a compression with a PSNR quality of 35 dB was used and 275 packets were sent through the radio module. The number of packets for other quality values can be extrapolated using Figure 9, where the number of packets after compressing the image and the PSNR values are linked.

Additionally, it was assumed that only one hop is done (from node to the BS) and no packets are lost during the transmission (there is no need for repetition).

As a conclusion, the proposed node acts as follows, according to the notes made above:

1. The LPM is woken up from its low-power mode and the MP is also woken up.
2. The MP captures and compresses an image from the CMOS camera with the selected PSNR.
3. The LPM requests fragments of the compressed image to the MP in order to send it through the RM. The MP sends fragments of a dummy file that has the same number of packets as obtained from Figure 9 for the selected PSNR.
4. When the image is completely sent, the LPM requires a shutdown for the MP, adopting its sleep power mode.

#### 4.2.3. Energy Analysis

**Figure 10:**
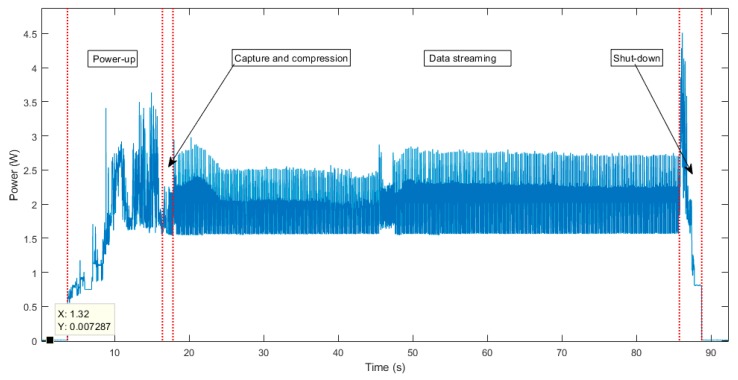
Power consumption of the proposed node capturing, compressing, and streaming an image.

Following the analysis described in the previous sections for a centralized processing approach, it was repeated iteratively (20 times), frequently obtaining similar results. As an example, Figure 10 shows the power consumption for one of these cases.

With the results obtained, a few conclusions can be observed. The mean energy consumption of the complete approach (image capture, compression, and transmission) is 43.90 mWh. As the image was compressed with a 35 dB quality and 275 packets were transmitted, the energy mean cost per packet is 0.1297 mWh. Also, it can be seen that the proposed node in a fully sleep mode consumes around 7.3 mW.

Another point to highlight is that, in this study, the time used to capture the image is much lower than the compression time (~11 times lower). In this sense, in the capture and compression phase, the energy used to capture an image with the CMOS camera can be disregarded, and it can be considered that this energy is just the result of the compression. Table 4 shows the mean energy cost for the different sub-processes.

### 4.3. Local Processing (Edge Computing Approach)

Local processing, despite the traditional approach in WSNs, consists of processing the data at the edge of the network (the nodes) and sending just the results of processing through the radio module to other nodes or to the upper layers of the network (if necessary). This study pretends to evaluate the energy cost of capturing an image from the CMOS camera of the proposed node, the local processing of it, and the transmission through the radio module of the results of its processing.

With this scheme, it is clear that the more complex the local processing, the more time it takes and the more energy will need for its execution. Therefore, the processing algorithm executed in the node is decisive in the final energy consumption analysis.

Taking this into account, no processing algorithm to be executed locally was selected. However, an estimation of consumption per processing time could be obtained for the proposed node. In this sense, a mean power consumption estimation is obtained. Thus, with this data and evaluating the execution time of the selected algorithm to be processed locally, the energy consumption can be obtained.

To obtain an estimation of the processing capabilities in terms of time of the proposed design, a CPU benchmark was conducted using the “sysbench” tool [54]. Thus, any other platform could be compared to the one proposed in terms of processing capabilities. The sysbench CPU test was executed using the default configuration and 20,000 as the maximum number to verify (sysbench --test=cpu--cpu-max-prime = 20,000 run). The result of the benchmark was an average of 44.4 ms per evaluation, giving 443.95 s of execution time.

#### Energy Analysis

**Figure 11:**
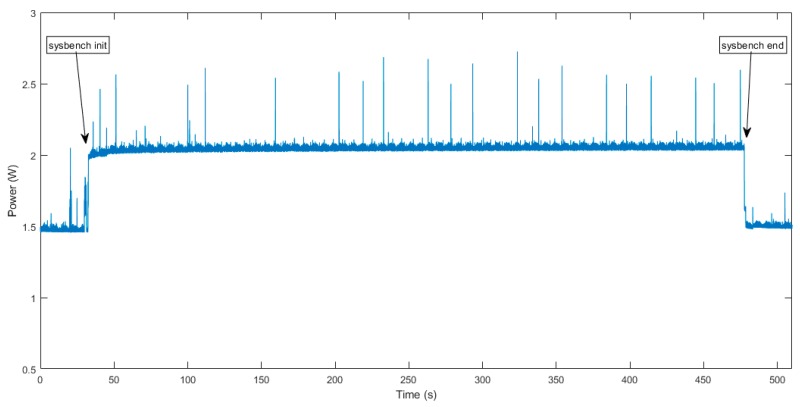
Power consumption at full processing.

To get the estimation of power consumption during processing and taking advantage of the analysis done before using the sysbench tool, in which a heavy processing was done, stressing the CPU, a mean power consumption of the proposed node at full load was obtained. In Figure 11, it is easy to note that the mean power consumption at full processing of the benchmark execution is 2.01 W.

## 5. Comparative Result Analysis

After conducting the tests, a few results can be obtained. Firstly, it can be said that for both study cases, the power-up and shut-down energy consumption will be the same in every cycle. Therefore, this energy cost can be evaluated as fixed for every MP cycle and can be estimated in 7.533 mWh. The image capture energy consumption, which is necessary in both scenarios, can be discarded because, in the test done in Section 4.2, this time is much lower than the compression time and cannot be easily measured.

An important point to highlight is that the compression needs much less energy than the data streaming, requiring 50.8 times more (with the compression used in Section 4.2.3). Thus, the slightly more time that the JPEG2000 algorithm consumes in comparison with the traditional JPEG is justified as the compression rate and quality are better. Furthermore, it needs to be said that the average compression time using the JPEG200 algorithm slightly differs between different compression ratios, and it can be considered as constant.

Also, in the streaming scenario, we can conclude that the energy consumption is principally attributable to the radio streaming (81.3% with the compression used in Section 4.2.3). Thus, the energy consumption directly depends on the image size after compression. In this sense, the energy consumption of an MP cycle using a data streaming schema ($E_{S}^{MP}$) can be obtained from the following equation (the meaning of each of its elements is detailed in Table 5):

(1) $$E_{S}^{MP}\left(mWh\right)=E_{W}+E_{C}+E_{P}\times N_{P}=8.235+0.1297\times N_{P}$$

Therefore, the energy consumption for the data streaming corresponds to a linear equation in which the number of transmitted packets shows the total amount of energy needed.

For the image processing at the edge, besides the energy consumption of the local processing, the radio transmission of the results must also be considered. This energy consumption can be estimated as the consumption of one packet transmission obtained in Section 4.2: 0.1297 mWh (although this estimation considers a full packet payload transmission and, sometimes, it could be lower). As has been said, in a local processing schema, the total energy consumption is going to be decided by the execution time of the local algorithm. In this sense, the energy used by the node in the algorithm processing phase ($E_{A}$) will be 2.01 Wh per hour of processing (or 0.5583 mWs/s). An equation for the energy consumption in a local processing schema ($E_{L}^{MP})$ similar to the streaming schema can be obtained:

(2) $$E_{L}^{MP}\left(mWh\right)=E_{W}+E_{P}+E_{A}\times t_{P}=0.9632+0.5583\times t_{P}$$

This is also a linear equation in which the energy consumption for the local processing schema is obtained using the processing time in seconds ($t_{P}$).

**Figure 12:**
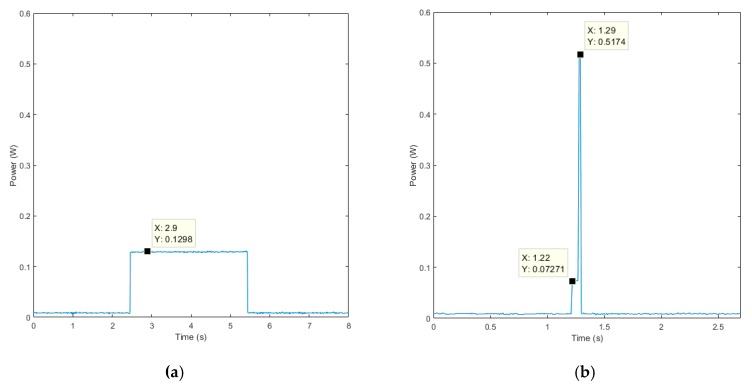
Power consumption for two LPM tasks: (**a**) wake-up from its sleep mode to attend its radio reception window; (**b**) wake-up from its sleep mode to acquire sensor data and transmit these data.

Furthermore, the energy consumption of the node when is in full sleep mode and when conducting its tasks must also be considered. These node tasks, apart from the MP actions, are the wake-up of the LPM in order to sensor data acquisition (and transmission when necessary) and to attend the communication on its reception window. Both tasks were characterized. In Figure 12a, the LPM wakes up and puts the RM in reception mode for three seconds to attend its reception window. In Figure 12b, the LPM wakes up from its sleep power mode, to acquire data from its sensors, and transmits these data.

The attending of the reception windows requires 0.108 mWh, while the sensor data and transmission require 3.7 μWh. In this sense, the energy consumption in a day for the proposed node will be (3) for the streaming schema and (4) for the local processing (the meaning of each of they elements is detailed in Table 6):

(3) $$\begin{array}{ll} E_{S}^{Node}\left(mWh/day\right) & =E_{S}^{MP}+E_{RM}^{LPM}+E_{S}^{LPM}+E_{LP}^{LPM}\\ & =\left(8.235+0.1297\times N_{P}\right)\times N^{MP}+0.108\times N_{W}^{LPM}\\ & +3.7\times 10^{-3}\times N_{S}^{LPM}+7.3\times 24 \end{array}$$

(4) ELNode(mWhday)=ELMP+ERMLPM+ESLPM+ELPLPM=(0.9632+0.5583×tP)×NMP+0.108×NWLPM+3.7×10−3×NSLPM+7.3×24

As the LPM’s processes can be applied to both schemas, the same granularity will result on the same amount of energy for both scenarios. In this sense, for both the image streaming and local processing, it was considered that the node needs to attend its reception window once per hour, and it needs to capture data from the traditional sensors four times per hour in order to simplify the analysis.

Taking this into account, different battery lifetime analyses were performed. These analyses were carried out using as a reference a standard lithium 18,650 cell with 12 Wh capacity.

As has been widely explained throughout this paper, the total energy consumption of the two approaches that are being analyzed depends on the compression ratio for the streaming scenario (number of packets transmitted) and the algorithm execution time for the local processing scenario. In this sense, the next graphs were obtained for different compression ratios and algorithm execution time. Also, different MP operations per day were evaluated.

**Figure 13:**
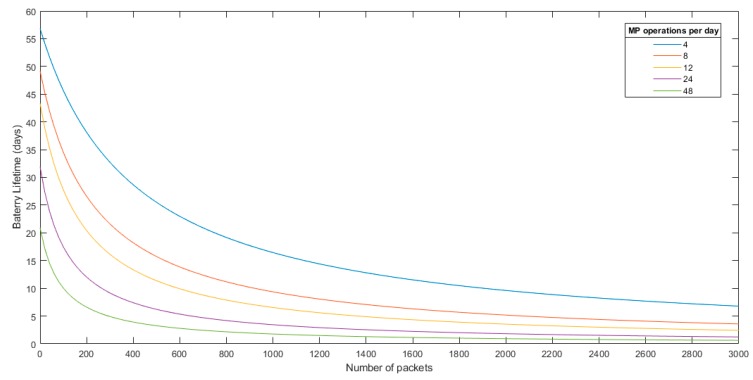
Battery lifetime analysis for the streaming schema.

**Figure 14:**
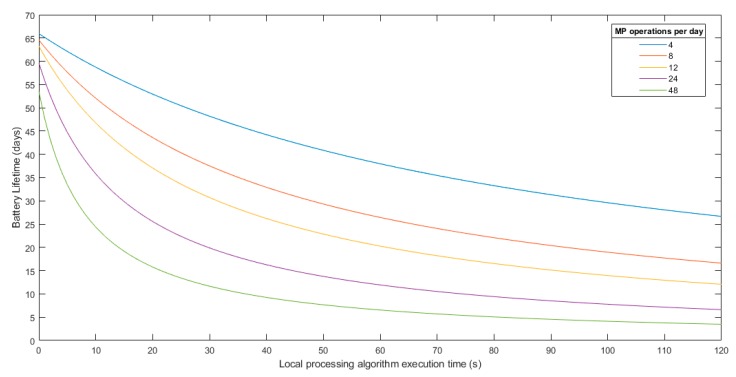
Battery lifetime analysis for the local processing schema.

Figure 13 shows the streaming scenario battery lifetime analysis based on the transmitted packets. Figure 14 shows the battery lifetime analysis for the local processing scenario based on the algorithm execution time.

Having this analysis, it is difficult to determine at first glance which approach is best. It is clear that in the streaming scenario, the image must have a good PSNR after decompression in order to process it in the upper layers (fog or cloud as example). The image quality threshold after decompression will depend on the application, but, normally, a PSNR after decompression in the range of 35–45 dB is a reasonable value in most applications for a post-decompression analysis.

For the local processing, it cannot be determined a priori how much execution time the algorithm will take. However, taking into account the high processing capabilities of the MP, supposing an execution time between 40 and 80 s is more than reasonable in a worst case analysis, even higher than what will be needed in most cases.

**Figure 15:**
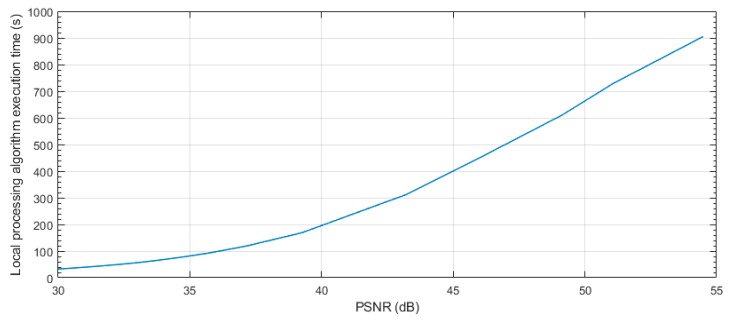
Local processing and data streaming bounder in function of PSNR and processing execution time.

In this sense, having the minimum PSNR value required or the execution time of the algorithm executed at the edge, the threshold value at which more efficient either to transmit the image or to process it locally can be determined (see Figure 15). This threshold can be calculated using Equations (1) and (2). Matching both equations and obtaining the relationship between number packets and PSNR (using Figure 9), this threshold value can be obtained.

**Figure 16:**
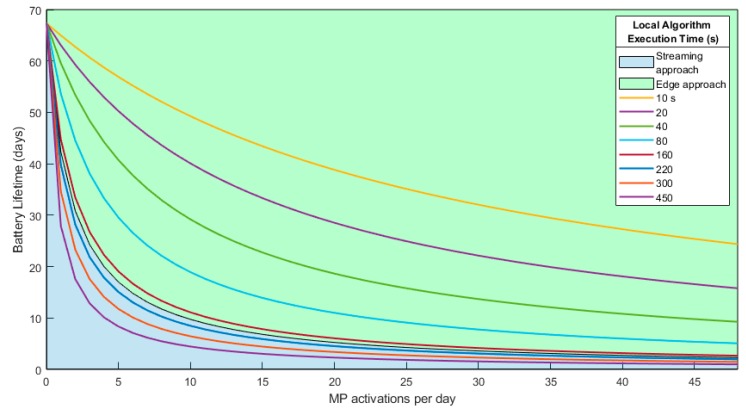
Local processing and data streaming bounder for a PSNR of 40 dB.

As can be seen in Figure 15, a centralized approach in which the image is transmitted through the radio module by the nodes and considering the application needs a minimum image quality of only 40 dB, the local processing algorithm execution time will need to be higher than 187.3 s to be worth transmitting the image. This case can be seen graphically in Figure 16, in which the battery lifetime was obtained for different MP activations per day. The lines in the green area correspond to the values in which local processing is more energy efficient. Any processing time higher than 187.3 s is in the blue area, in which it is better to transmit the image. If the image is transmitted through the radio module, the battery lifetime will be the limit of both areas. Using this figure, it is easy to identify the border from each approach or when one is better approach than or another (see each area), depending on the execution time of the local algorithm.

Having the high processing capabilities of the MP, the local algorithm needs to be quite complex to reach this execution time. To give an example, an algorithm currently being developed by the authors to predict and detect forest fires using the proposed platform takes just 4 s.

In this sense, it is easy to note that the local processing is much more energy efficient in most of the practical cases. Just when the processing algorithm will require a high execution time that the energy used results higher than the energy of transmitting the image, it could be considered to transmit the image.

In addition, in this analysis, we are assuming a one-hop schema; if more than a hop is needed to reach the destination, more energy will be used by the network.

Also, it is important to point out that local processing will analyze the image in a raw format having no losses, while in the streaming scenario, the image will have certain losses that may bring about an unusable result. Furthermore, with the reprogramming capabilities of the proposed node, it is simple to change and improve the processing algorithm remotely.

Additionally, the radio module configuration in the study conducted for the data streaming scenario was set up to its maximum transmission ratio. It needs to be said that with this configuration, the lower possible radio range is achieved, obtaining just a hundred meters of coverage. In an ideal scenario, having line of sight and a very short distance between the nodes, this configuration of the radio module could be valid. Normally, however, this configuration may not be enough, requiring lower data transmission ratios and increasing the radio range, which will result in larger transmission times and, therefore, larger energy consumption per packet.

## 6. Conclusions

In this paper, we analyzed the main issues/constraints that WMSN has nowadays; that is, computational resources, radio communications, sensor integration and management, and energy consumption. On the basis of these problems, a complete WMSN node architecture was designed.

The proposed node offers a high integration and variety of sensors (both multimedia and scalar) for different environmental applications. Additionally, the device is able to make use of data fusion algorithms in order to obtain more complete and better results. Furthermore, the node is easily adaptable, by adding or removing parts, depending on the final application. Also, thanks to the reprogramming capabilities of the node, new firmwares can be easily updated remotely, making the upgrade and scalability of the network simpler, facilitating the work of scientists and biologists. All those features make the proposed node a multipurpose platform that is capable of adapting to different environmental scenarios and research fields.

The combination of the ultra-low power microcontroller (LPM) and the multimedia processor (MP) offers many advantages in terms of computing capabilities and energy management, offering a great tradeoff between processing capabilities and battery lifetime. The MP and the multimedia sensors act as additional resources of the node and could be enabled and used just when necessary depending on the environmental conditions and on the state of charge. Thanks to this, different sampling rates can be used for both the scalar sensors and the multimedia sensors. Additionally, having the high processing capabilities of the MP, processing of multimedia data can be performed at the edge, reducing energy consumption and extending battery lifetime. In this sense, the multiprocessor architecture offers a great tradeoff between processing capabilities and energy consumption.

The proposed platform is not only a theoretical design. A network using the proposed device was developed in the Doñana National Park. With this real deployment, we discover that this environment could be very tough, with very high temperatures. Thanks to the design of the platform and its processing scheme, however, it demonstrates that it can work continuously without issues for a year. A proof of the effectiveness of the system and the energy management is the fact that none of the 10 nodes has fully depleted its battery during the entire deployment.

In addition, taking into account that energy consumption is a critical constraint in WMSNs, an energy analysis between data streaming and edge processing was performed using the proposed node. Both scenarios were fully characterized, obtaining the energy consumption for each processing step. On the basis of the obtained results, battery lifetime estimations were obtained for both approaches. Thanks to this, a work environment for energy characterization was set up, allowing us to do future studies.

After characterizing both approaches, it was seen that different parameters affect both scenarios (number of packets and execution time), resulting in different energy consumptions, making it quite difficult to decide which approach is the best. In this sense, a way to obtain when it is more efficient either to transmit the image or process it locally based on the needed PSNR or the local execution time was obtained. Also, an example in which a minimum PSNR of 40 dB requires that the execution time of a local algorithm be greater than 187.3 s was provided. Taking this analysis into account and thanks to the tradeoff between processing capabilities and energy management of the proposed node, it was obtained that, in most applications, edge processing is more efficient than the traditional WMSN approach, where the information is sent and processed later in a central node (centralized processing).

## Figures and Tables

**Table 1:** Cost detail for the basic node parts.

Part	Price
PCB Board	1.20 €
Electronic components	21.29 €
LoRa Antenna	6.21 €
Assembly	3.00 €
Enclosure	10 €
Total	41.70 €

**Table 2:** Cost detail for the different additions that can be added to the proposed system. MP, multimedia processor.

Part	Price
MP (Raspberry Pi 3)	34.97 €
CMOS Camera	20.05 €
Thermal Camera	238.20 €
Weather Station	52.18 €
Stepper Motor	4.07 €

**Table 3:** Proposal of three different configurations.

Configuration A	Price	Configuration B	Price	Configuration C	Price
Node basis	41.70 €	Node basis	41.70 €	Node basis	41.70 €
Weather Station	52.18 €	MP	34.97 €	MP	34.97 €
		CMOS Camera	20.05 €	CMOS Camera	20.05 €
				Stepper Motor	4.07 €
				Weather Station	52.18 €
Total	93.88 €	Total	96.72 €	Total	152.97 €

**Table 4:** Energy cost for the different sub-processes when streaming an image.

Sub-Process	Energy Cost
MP power-up	6.13 mWh
Image capture and compression	0.702 mWh
Packet transmission	35.671 mWh
MP shut-down	1.403 mWh

**Table 5:** Description of the parameters used in Equations (1) and (2).

Parameter	Description
*E_W_*	Energy used for the MP to power-up and shut-down
*E_C_*	Energy for the compression
*E_P_*	Energy per packet transmitted
*E_A_*	Energy used by the processing algorithm
*N_P_*	Number of packets transmitted
*t_p_*	Time used by the processing algorithm

**Table 6:** Description of the parameters used in Equations (3) and (4).

Parameter	Description
$E_{S}^{Node}$	Energy used per day by the node using the data streaming schema
$E_{S}^{MP}$	Energy used per day for the MP cycles using the data streaming schema
$E_{RM}^{LPM}$	Energy used per day to attend the radio reception window
$E_{S}^{LPM}$	Energy used per day to read the scalar sensors
$E_{LP}^{LPM}$	Energy used per day by the node in its fully sleep low power mode
$N^{MP}$	Number of actions realized per day with the MP
$N_{W}^{LPM}$	Number of times per day that the node must attend its reception window.
$N_{S}^{LPM}$	Number of times per day that the node has collect data from the scalar sensors.
$E_{L}^{Node}$	Energy used per day by the node using the local processing schema
$E_{L}^{MP}$	Energy used per day for the MP cycles using the local processing schema
